# Identifying DNA-binding proteins by combining support vector machine and PSSM distance transformation

**DOI:** 10.1186/1752-0509-9-S1-S10

**Published:** 2015-02-06

**Authors:** Ruifeng Xu, Jiyun Zhou, Hongpeng Wang, Yulan He, Xiaolong Wang, Bin Liu

**Affiliations:** 1 School of Computer Science and Technology, Harbin Institute of Technology Shenzhen Graduate School, Shenzhen, Guangdong, China; 2 Key Laboratory of Network Oriented Intelligent Computation, Harbin Institute of Technology Shenzhen Graduate School, Shenzhen, Guangdong, China; 3 School of Engineering & Applied Science, Aston University, Birmingham, UK

**Keywords:** DNA-binding protein, position specific score matrix, support vector machine, distance transformation

## Abstract

**Background:**

DNA-binding proteins play a pivotal role in various intra- and extra-cellular activities ranging from DNA replication to gene expression control. Identification of DNA-binding proteins is one of the major challenges in the field of genome annotation. There have been several computational methods proposed in the literature to deal with the DNA-binding protein identification. However, most of them can't provide an invaluable knowledge base for our understanding of DNA-protein interactions.

**Results:**

We firstly presented a new protein sequence encoding method called PSSM Distance Transformation, and then constructed a DNA-binding protein identification method (SVM-PSSM-DT) by combining PSSM Distance Transformation with support vector machine (SVM). First, the PSSM profiles are generated by using the PSI-BLAST program to search the non-redundant (NR) database. Next, the PSSM profiles are transformed into uniform numeric representations appropriately by distance transformation scheme. Lastly, the resulting uniform numeric representations are inputted into a SVM classifier for prediction. Thus whether a sequence can bind to DNA or not can be determined. In benchmark test on 525 DNA-binding and 550 non DNA-binding proteins using jackknife validation, the present model achieved an ACC of 79.96%, MCC of 0.622 and AUC of 86.50%. This performance is considerably better than most of the existing state-of-the-art predictive methods. When tested on a recently constructed independent dataset PDB186, SVM-PSSM-DT also achieved the best performance with ACC of 80.00%, MCC of 0.647 and AUC of 87.40%, and outperformed some existing state-of-the-art methods.

**Conclusions:**

The experiment results demonstrate that PSSM Distance Transformation is an available protein sequence encoding method and SVM-PSSM-DT is a useful tool for identifying the DNA-binding proteins. A user-friendly web-server of SVM-PSSM-DT was constructed, which is freely accessible to the public at the web-site on http://bioinformatics.hitsz.edu.cn/PSSM-DT/.

## Introduction

DNA-binding proteins are pivotal to the cell functions such as DNA replication, transcriptional regulation, packaging recombination, DNA repair, DNA modification and other fundamental activities associated with DNA. For example, in eukaryotic cells, histones which is a typical type of DNA-binding protein often help package chromosomal DNA into a compact structure, and as another typical DNA-binding protein, restriction enzymes are DNA-cutting enzymes found in bacteria that recognize and cut DNA only at a particular sequence of nucleotides to serve a host-defense role. DNA-binding proteins represent a broad category of proteins, known to be highly diverse in sequence and structure. Structurally, they have been divided into eight structural groups, which were further classified 54 protein structural families[1,2]. Functionally, protein-DNA interactions play various roles across the entire genome as previously mentioned [3]. The past decade has witnessed tremendous progress in genome sequencing [4-7]. According to the Genome On Line Database, the complete sequenced genomes of almost 1000 cellular organisms have been released, and about 5000 active genome sequencing projects are on the way [8,9]. The unprecedented amount of genetic information has provided hundreds of thousands of protein sequences [10], indicating that a challenging problem to elucidate their functions is posed.

At present, several experimental techniques have been employed for identifying DNA-binding proteins, such as filter binding assays, genetic analysis, chromatin immunoprecipitation on microarrays, and X-ray crystallography. But experimental approaches for identifying the DNA-binding proteins are costly and time consuming. It would be highly desirable to develop computational approaches that can automatically determine whether a novel sequence binds to DNA or not. Therefore, a reliable identification of DNA-binding proteins with effective computational approach is an import research topic in the proteomics fields. It has been observed that many attempts have been made for identifying DNA-binding proteins and many effective computational predicting methods have been proposed for analyzing it in the literature. The computational methods represent a broad category of predicting methods for DNA-binding proteins, known to be highly diverse in classifiers and protein representation.

In terms of classifiers, the computational methods can be divided into template-based and machine-learning-based methods, depending on how they use the information from the putative DNA-binding proteins. Template-based methods can be further classified into two classes, one of which utilize a structural comparison protocol to detect significant structural similarity between the query and a template known to bind DNA at either the domain or the structural motif to assess the DNA-binding preference of the target sequence [11,12] and the other employ a sequence comparison protocol (such as PSI-BLAST) to detect significant sequence similarity between the query and a template known to bind DNA to evaluate the DNA-binding preference of the target sequence [13]. Machine-learning-based methods do not perform direct structural comparison, but typically follow a machine-learning framework. To obtain good predictive model, various machine-learning algorithms have been employed to construct classification models, such as support vector machine (SVM) [14-17], neural network [18-22], random forest [23], naïve Bayes classifier [24,25], nearest neighbor [26] and ensemble classifiers [27,28], [29]

In the task of computational protein function prediction, there are two major problems: choice of the classification algorithm and choice of the protein representation. Depending on the choice of protein representation, these computational predictive methods can be classified into two categories: i) analysis from protein structure [19,20,28,30] and ii) prediction from amino acid sequence[11,21,31-33]. In case of structure-based prediction methods, Stawiski et al. [19] examined positively charged patches on the surface of putative DNA-binding proteins in comparison with that on non DNA-binding proteins. They employed 12 features including the patch size, hydrogen-bonding potential, and the fraction of evolutionary conserved positively charged residues and other properties of the protein to train a neural network (NN) for identifying DNA-binding proteins. Ahmad and Sarai [20] trained a NN classifier using three features, including net charge, electric dipole and quadruple moments of the protein. Bhardwaj et al. [15] examined the sizes of positively charged patches on the surface of putative DNA-binding proteins. They based their SVM classifier on the protein's overall charge, overall and surface amino acid composition. Szilágyi and Skolnick [34] previously trained a logistic regression classifier using the amino acid composition, the asymmetry of the spatial distribution of specific residues and the dipole moment of the protein. Guy Nimrod and Andras Szilágyi et al. [23] recently developed a random forest classifier based on the electrostatic potential, cluster-based amino acid conservation patterns and the secondary structure content of the patches, as well as features of the whole protein including its dipole moment. Since the negative samples are much more than real DNA-binding proteins, this is an imbalanced binary classification problem from the view of machine learning. Song et al. [35] employed ensemble classifier [36] to solve this problem and improved the identification. Several methods considering the sequence-order effects were proposed, and the experimental results showed that this information can improve the predictive performance [37,38].

The accuracy of structure-based prediction methods is usually higher, but they can't be used in high throughput annotation, as it requires the high-resolution 3D structure of the query sequence. Until now, many computational methods have been proposed for identifying DNA-binding protein from their amino acid sequences directly. There are four different categories of protein sequence features and three kinds of sequence encoding methods have been proposed [31,39-41]. The four categories of features are composition information, structural and functional information, physicochemical properties and evolutionary information and the three kinds of coding methods are overall composition-transition-distribution called OCTD (Global method), autocross-covariance (ACC) transformation (Nonlocal method) and split amino acid (SAA) Transformation (Local method). A comprehensive survey of these methods can be found in related research work [42-44]. However, most of the present encoding methods provided limited information to explain the mechanisms of DNA-protein interactions. It is desirable to explore a novel encoding method that can reveal the binding mechanism of DNA-proteins interactions.

In the current study, to further advance the prediction accuracy and understand the binding mechanism of DNA-protein interaction, we presented here a novel encoding method called PSSM distance transformation (PSSM-DT) to transform the PSSM profiles of query sequences into uniform numeric representations. Then we constructed a DNA-binding protein identification method SVM-PSSM-DT by combining the PSSM-DT with SVM. The benchmark test and independent test showed that PSSM-DT is a promising protein encoding method.

## Methods

As shown by a series of recent publications [45-59] and summarized in a comprehensive review, to develop a useful statistical prediction method or model for a biological system, one needs to engage the following procedures: (i) construct or select a valid benchmark dataset to train and test the predictor; (ii) formulate the samples with an effective mathematical expression that can truly reflect their intrinsic correlation with the target to be predicted; (iii) introduce or develop a powerful algorithm (or engine) to operate the prediction; (iv) properly perform cross-validation tests to objectively evaluate the anticipated accuracy of the predictor; (v) construct a web-server for the prediction method. Below, we describe our proposed method followed such a general procedure.

### Dataset

To construct a high quality benchmark dataset, only experimentally confirmed data were collected. The benchmark dataset S can be formulated as

(1) $$\text{S = }{\text{S}}^{+}\cup {\text{S}}^{-}$$

where the subset S^+ ^contains 525 DNA-binding proteins, the subset S^- ^consists of 550 non DNA-binding proteins and the symbol ∪ represents the "union" in the set theory. The benchmark dataset was obtained according to the following procedure. (**1**) Extract DNA-binding protein sequences from Protein Data Bank (PDB) released at December 2013 by searching the mmCIF keyword of 'DNA binding protein' through the advanced search interface. (**2**) Remove the sequences with length of less than 50 amino acid residues and character of 'X'. (**3**) Utilize PISCES to cutoff those sequences that have >= 25% pairwise sequence identity to any other in the same subset. Thus the subset S^+ ^consisting 525 sequences is obtained. (**4**) Randomly extract some non DNA-binding proteins from Protein Data Bank, then utilize PISCES to cutoff those sequence that have >= 25% pairwise sequence identity to any other in the same subset and remove all the sequences with less than 50 amino acids or with character of 'X'. Thus the subset S^- ^containing 550 sequences is obtained. A complete list of all the PDB codes and sequence for the benchmark dataset can be found in Supporting Information S1.

### Position Specific Scoring Matrix

Evolutionary information, one of the most import kinds of information in protein functionality annotation in biological analysis, has been widely used in many studies [21,60-63]. In this study, evolutionary information in forms of PSSM profile of every protein sequence is obtained by running the PSI-BLAST [64] program to search the non-redundant (NR) database through three iteration with 0.001 as the E-value cutoff for multiple sequence alignment. The final PSSM profile is a matrix with dimension of L*20 (excluding dummy residue X), which can depicted as follows:

(2) $$\text{PSS}M=\left[\begin{array}{cccc} S_{1,1}^{} & S_{1,2}^{} & \cdots \; & S_{1,20}^{}\\ S_{2,1}^{} & S_{2,2}^{} & \cdots \; & S_{2,20}^{}\\ \vdots & \vdots & \vdots & \vdots \\ S_{L,1}^{} & S_{L,2}^{} & \cdots \; & S_{L,20}^{} \end{array}\right]$$

where *L *is the length of protein, the *S*_*i*,*j *_represents the occurrence probability of amino acid *j *at position *i *of the protein sequence, the rows of matrix represent the positions of the sequence and the columns of the matrix represent the 20 types original amino acids. PSSM scores are generally shown as positive or negative integers. Positive scores indicate that the given amino acid occurs more frequently in the alignment than expected by chance, while negative scores indicate that the given amino acid occurs less frequently than expected. Large positive scores often indicate critical functional residues, which may be active site residues or residues required for other intermolecular interactions. Therefore the element of PSSM profile can be used to approximately measure the occurrence probability of the corresponding amino acid at a specific position.

### PSSM distance transformation

It has been reported that dipeptides containing two residues separated by a distance along the sequence are important for protein functionality annotation in the work [65]. Additionally, the PSSM score can approximately measure how frequently an amino acid occurs at a position of a sequence. Accordingly, we present here a PSSM distance transformation (PSSM-DT) method to encode the feature vector representation from the PSSM information. PSSM-DT can transform the PSSM information into uniform numeric representation by approximately measuring the occurrence probabilities of any pairs of amino acid separated by a distance along the sequence in a sequence. PSSM-DT results in two kinds of features: PSSM distance transformation of pairs of same amino acids (PSSM-SDT) and PSSM distance transformation of pairs of different amino acids (PSSM-DDT). The PSSM-SDT features approximately measure the occurrence probabilities of pairs of same amino acids separated by a distance of *lg *along the sequence in a sequence, which can be calculated as below

(3) $$\text{PSSM - SDT(}i,lg\text{) = }\overset{L-lg}{\underset{j=1}{\sum }}S_{i,j}*S_{i,j+lg}/\left(L-lg\right)$$

where *i *is one type of the amino acid, *L *is the length of the sequence, *S_i,j _*is the PSSM score of amino acid *j *at position *i*. In such a way, 20*LG is the number of PSSM-SDT features, where *LG *is the maximum value of *lg *(*lg *= 1, 2,...,*LG*).

The PSSM-DDT features approximately measures the occurrence probabilities of pairs of different amino acids separated by a distance of *lg *along the sequence, which can be calculated by:

(4) $$\text{PSSM - DDT(}i1,i2,lg\text{) = }\overset{L-lg}{\underset{j=1}{\sum }}S_{i1,j}*S_{i2,j+lg}/\left(L-lg\right)$$

where *i*1 and *i*2 refer to two different types amino acids. Similarly, the total number of PSSM-DDT features can be calculated as 380*LG.

PSSM-DT is the combination of variable PSSM-SDT and PSSM-DDT. Thus a sequence can be transformed into a uniform feature vector with a fixed dimension of 400*LG by using variable PSSM-DT from its PSSM profile.

### Support vector machine

Support vector machine is a machine learning algorithm based on statistical learning theory presented by Vapnik (1998) [66], which uses a non-linear transformation to map the input data to a high dimensional feature space where linear classification is performed. It is equivalent to solving the quadratic optimization problem:

(5) $${\overset{}{\underset{w,b,\xi_{i}}{\text{min}}}}^{}\frac{1}{2}w*w+C\underset{i}{\sum }\xi_{i}$$

(6) $$\begin{array}{c} \text{s}\text{.t}\text{. }{\text{y}}_{i}\left(\phi \left(x_{i}\right)*w+b\right)\geq 1\text{ - }\xi_{\text{i}},i=1,...,m,\\ \;\xi_{i}\geq 0,i=1,...,m, \end{array}$$

Where *x_i _*is a feature vector labeled by *y_i _*∈ {-1, +1} and *C*, called the cost, is the penalty parameter of the error term. The above model called soft-margin SVM can be able to tolerate noise within the data, which analyze an example by generating a separating hyper-plane with *f*(*x*) = *ϕ*(*x*)·*w *+ *b *= 0. Through resolving the above model with lagrangian multiplier method, we obtain $w=\sum_{j}\alpha_{h}*y_{j}*\phi \left(x_{j}\right)$ and $w\cdot \phi \left(x_{i}\right)=\sum_{j}\alpha_{h}*y_{j}*\phi \left(x_{j}\right)\cdot \phi \left(x_{i}\right)$, which provides an efficient approach to solve SVM without the explicit use of the non-linear transformation, where $K\left(x_{i},x_{j}\right)=\phi \left(x_{i}\right)\cdot \phi \left(x_{j}\right)$is the kernel function. Application of SVM in bioinformatics problems has been widely explored [15,67-69]. At present, the publicly available LIBSVM, which take the radial basis function (RBF) as the kernel function, is employed as the implementation of SVM. RBF is defined as below

(7) $$K\left(X_{i},X_{j}\right)=\text{exp}\left(-\gamma {∥X_{i}-X_{j}∥}^{2}\right)$$

In this study, SVM parameter *γ *and penalty parameter *C *were optimized based on 5-fold cross validation in a grid-based manner with respect to the sequence in benchmark dataset. In this study, jackknife test is taken as the evaluation method to calculate the evaluation criteria. For a dataset with *N *sequences, each time, one of sequence is taken out as testing sequence and the remaining sequences are employed as training dataset. This process repeated until each sequence in the dataset is tested exactly once. The average performance over all the processes is taken as the final results.

### Evaluation metrics

Sensitivity (SN), Specificity (SP), Accuracy (ACC), Matthews Correlation Coefficient (MCC), Receiver Operating Characteristic (ROC) curve and the area under ROC curve (AUC) are employed in this work. All of the above measurements were calculated in the case of jackknife validation and defined as follows:

(8) SN = TP/(TP+FN)

(9) SP = TN/(TN+FP)

(10) ACC = (TP+TN)/(TP+FP+TN+FN)

(11) $$\text{MCC = (}TP*TN-FP*FN\text{)/}\sqrt{\left(TP+FN\right)*\left(TP+FP\right)*\left(TN+FP\right)*\left(TN+FN\right)}$$

In this study, TP, FP, TN and FN donated the numbers of true positives, false positives, true negatives and false negatives, respectively. ACC denotes the percentage of both positive instances and negative instances correctly predicted. SN and SP represent the percentage of positive instances correctly predicted and that of negative instances correctly predicted, respectively. A ROC curve is a plot of Sensitivity versus (1-Specificity) and generated by shifting the decision threshold. AUC gives a measure of classifier performance. An AUC of 1.0 indicates perfect classifier whereas an AUC of classifier no better than random is 0.5. The value of MCC measures the degree of overlap between the predicted labels and true labels of all the samples in the benchmark dataset. It returns a value between -1 and +1. A perfect prediction at 100% accuracy yields a MCC of +1, whereas a random prediction gives a MCC of 0 and a terrible prediction at 0 accuracy produce a MCC of -1.

## Results and discussion

### The selection of *LG *and features

**Figure 1:**
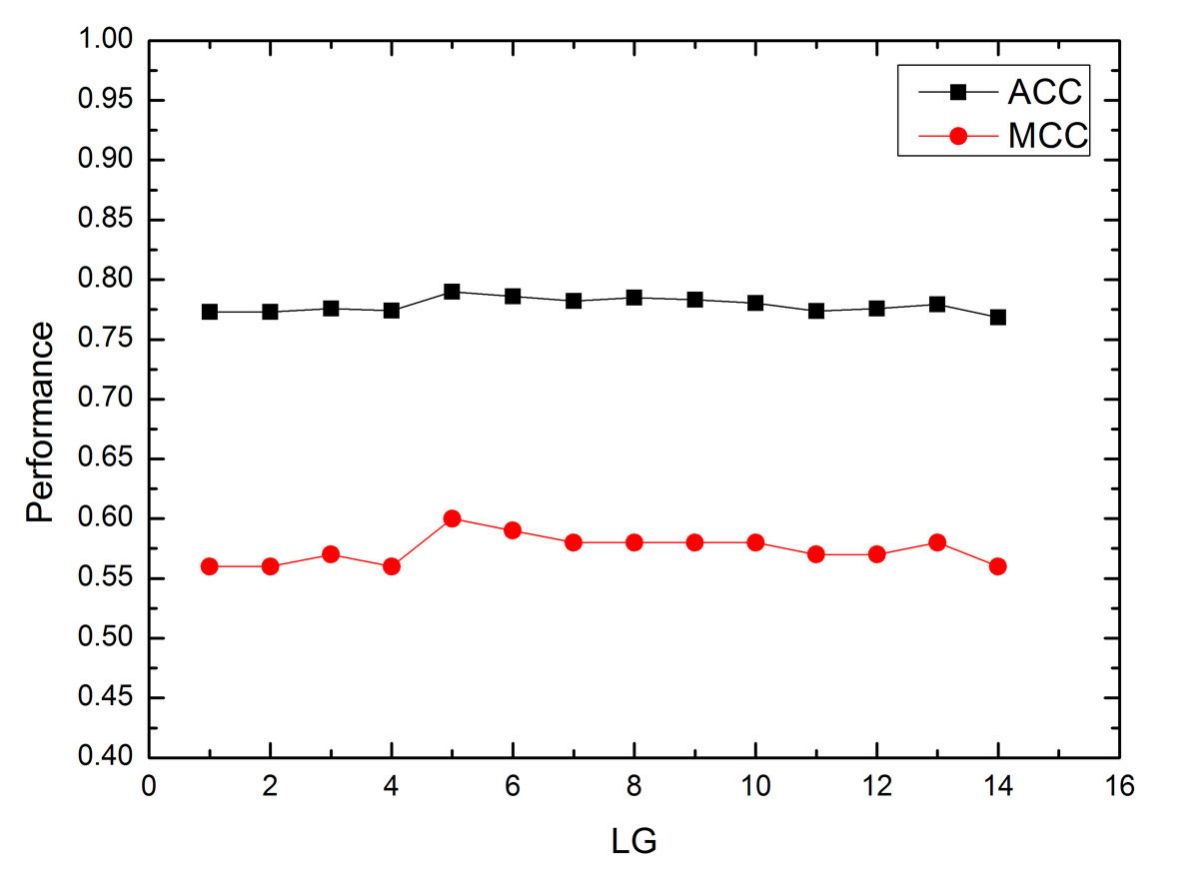
**The performance of SVM-PSSM-DT with different *LG***. *LG *is a parameter in the present method SVM-PSSM-DT. The average ACC and MCC values were used to evaluate the impact of different *LG *values on the performance of SVM-PSSM-DT. The results were got by testing the model on the benchmark dataset by five-fold-cross-validation.

To evaluate the PSSM-DT method, we analyzed the impact of parameter *LG *on the predictive performance of the proposed model. The predictive results of SVM-PSSM-DT with different values of *LG *on the benchmark dataset by using five-fold cross validation is shown in Figure 1. As can be seen from the Figure, the value of *LG *has modest impact on both the ACC and MCC metrics. The ACC firstly increases to a maximum value and then slightly goes down as the *LG *value increases. So we can conclude that SVM-PSSM-DT achieves the best performance when *LG *= 5, which mean that the dimension of the feature space applied in this work is 2000. Therefore, the parameter *LG *was set as 5 for the following experiments.

In this study, we proposed three protein representations, including PSSM-DT, PSSM-SDT and PSSM-DDT. Table 1 lists predictive results of the three proposed protein representation according to jackknife validation on benchmark dataset. As a result, the predictor using PSSM-DT yields the highest ACC of 79.96%, MCC of 0.622 and AUC of 86.50%. So in the following experiments, the PSSM-DT based representation was applied to encode the features from PSSM profile.

**Table 1:** Results on benchmark dataset of different features through jackknife validation.

Methods	Acc(%)	MCC	SN(%)	SP(%)
PSSM-DDT^a^	79.72	0.607	81.33	78.18
PSSM-SDT^b^	74.79	0.512	77.147	74.93
PSSM-DT^c^	79.96	0.622	81.91	78.00

PSSM-DT can extract two kinds protein features, called PSSM-DDT and PSSM-SDT respectively. And PSSM-DT represents the combination of PSSM-DDT and PSSM-SDT. The results were got by testing the models on benchmark dataset through jackknife validation.

^a^the predictor using PSSM-DDT as protein representation

^b^the predictor using PSSM-SDT as protein representation

^c^the predictor using PSSM-DT as protein representation

### Feature analysis

To further investigate the importance of the features and reveal the biological meaning of the features in PSSM-DT, we followed the study [50,70,71] to calculate the discriminant weight vector in the feature space. The sequence-specific weight obtained from the SVM training process can be used to calculate the discriminant weight of each feature to measure the importance of the features. Given the weight vectors of the training set with *N *samples obtained from the kernel-based training **A **= [*a*_1_, *a*_2_, *a*_3_,...,*a_N_*], the feature discriminant weight vector **W **in the feature space can be calculated by the following equation:

(12) $$W=A\cdot M={\left[\begin{array}{c} a_{1}\\ a_{2}\\ \vdots \\ a_{N} \end{array}\right]}^{T}\left[\begin{array}{cccc} m_{11} & m_{12} & \cdots \; & m_{1j}\\ m_{21} & m_{22} & \cdots \; & m_{2j}\\ \vdots & \vdots & \ddots & \vdots \\ m_{N1} & m_{N2} & \cdots \; & m_{Nj} \end{array}\right]$$

where **M **is the matrix of sequence representatives in PSSM-DT; **A **is the weight vectors of the training samples; *N *is the number of training samples; *j *is the dimension of the feature vector. The element in **W **represents the discriminative power of the corresponding feature.

**Figure 2:**
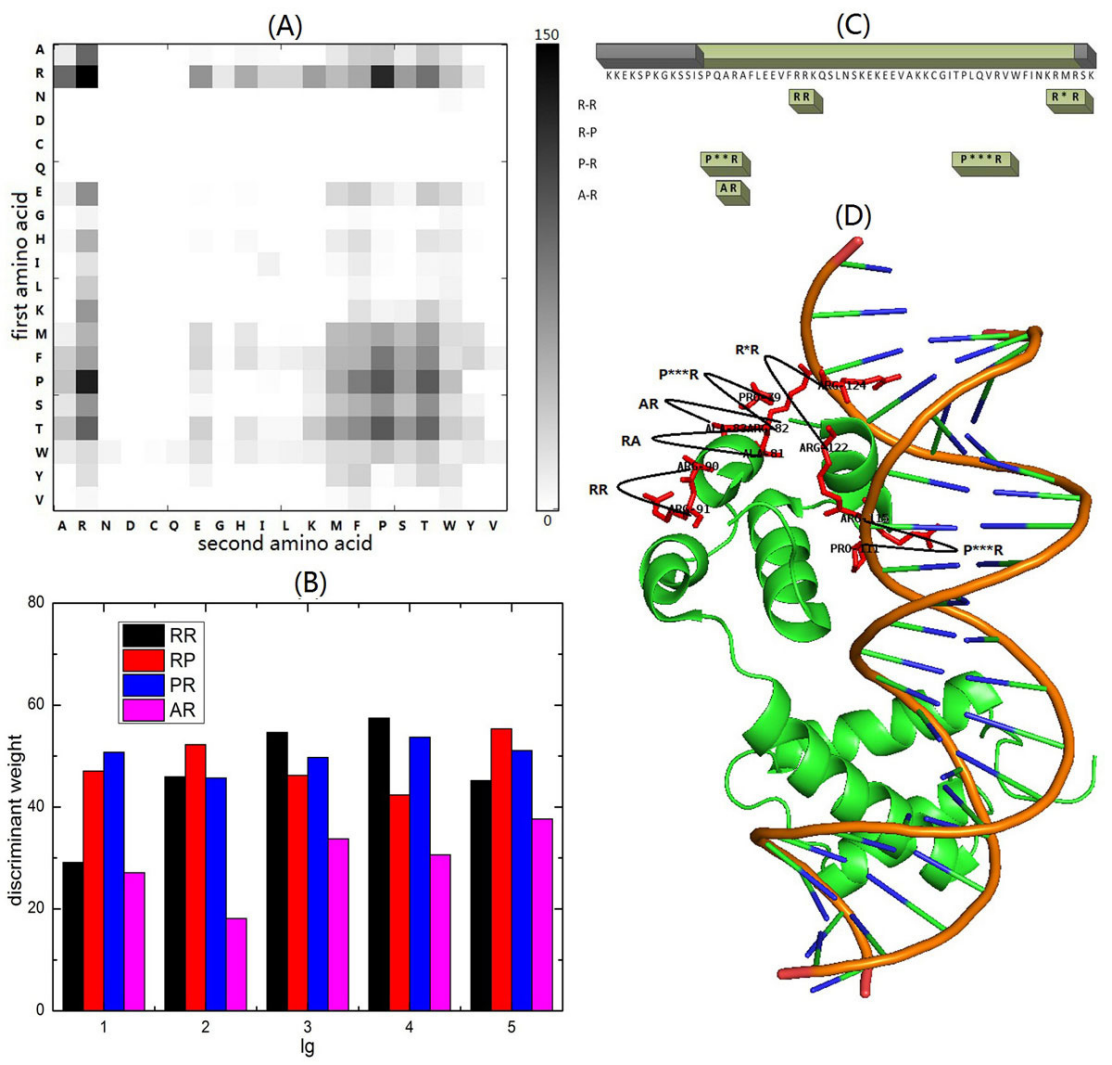
**Feature analysis on protein **1AKH**chain A**. (A) The discriminant weights of the 400 amino acid pairs. Each element in the figure refers to the quadratic sum of the discriminant weights of descriptors with positive discriminant weight for a certain amino acid pair. A amino acid pair is identified by two amino acids, the x-axis and y-axis represent its second amino acid and first amino acid, respectively. (B) The discriminant weights of the descriptors with different *lg *values for the top four most discriminant amino acid pairs, including pair(R,R), pair(R,P), pair(P,R) and pair(A,R). (C) The occurrence distributions of the descriptors for the top four most discriminant amino acid pairs on the DNA-binding regions and non DNA-binding regions of protein 1AKH chain A, respectively. The regions in green color are non DNA-binding regions and the region in grey color is DNA-binding protein. (D) The occurrence distributions of the descriptors for the top four most discriminant amino acid pairs on the three dimensional structure of protein 1AKH chain A. The green sections are the three dimensional structure of protein and the brown sections are the three dimensional structure of the DNA.

In this study, we are only interested in the descriptors frequently occurring in positive samples (DNA-binding proteins). Therefore, the discriminant weight of an amino acid pair is calculated as the quadratic sum of the discriminant weights of the corresponding descriptors with positive discriminant weight for this amino acid pair. The discriminant weights of all the 400 amino acid pairs in PSSM-DT are depicted in Figure 2A. According to this figure, the top four most discriminative amino acid pairs are (R, R), (R, P), (P, R) and (A, R), which indicate that the amino acid R (Arg) and A (Ala) are important for identifying the DNA-protein interaction. This conclusion is consistent with Szilágyi and Skolnick's study [34], in which they found that the percentage of Arg, Ala, Gly, Lys and Asp are useful for identification of DNA-binding proteins. Sieber and Allemann [72] found that R (348) can't directly interact with the nucleobases, but can determine the DNA binding specificity of the basic helix-loop-helix proteins (BHLH) E12 by directly interacting with both the phosphate backbone and the carboxylate of E(345) resulting in locking the side chain conformation of E(345). what's more, by comprehensively analyzing the three dimensional structures of protein-DNA complexes, Rohs and West et al. [73] demonstrated that the binding of R to narrow minor grooves can be applied to mode for protein-DNA recognition, indicating that R is an important component in protein-DNA binding activity. It has been previously reported that the DNA usually enveloped with negative electrostatic potential and the amino acid R shows positive charge [12], which explain the reason why the amino acid R is important for DNA-binding protein identification.

The discriminant weight of the descriptors for pairs (R, R), (R, P), (P, R) and (A, R) with different *lg *values are shown in Figure 2B. As indicated by the figure, the descriptor with *lg *of 4 for pair (R, R) has the highest discriminant power. For pair (R, P) and (P, R), the discriminant weight of all descriptors are slightly different. In case of pair (A, R), the descriptor with *lg *of 5 is the most discriminative feature. In conclusion, for an amino acid pair, the distance between the two amino acids along the sequence can impact its discriminant power in DNA-binding protein identification.

Additionally, we take protein 1AKH [PDB:1AKH] chain A as an example to show the availability of PSSM-DT based protein representation on DNA-binding protein identification. 1AKH is known as the MATa1/MATα2 homeodomain heterodimer and its chain A is the yeast mating type transcription factors (MATa1). MATa1 proteins are members of the homeodomain superfamily of DNA-binding proteins and contact the DNA with its homeodomain. It always folds into a compact three-helix domain containing a helix-turn-helix DNA-binding motif. Figure 2C lists the distributions of descriptors for the top four most discriminative pairs on the sequence of MATa1 protein. From this figure we can see that there are 5 occurrences of the proposed descriptors in the DNA-binding region and no occurrence in the non DNA-binding regions. There are totally 5 descriptors occurred in the DNA-binding region, including pair(R, R) with *lg *of 1, pair(R, R) with *lg *of 3, pair(P, R) with lg of 2, pair(P, R) with *lg *of 3 and pair(A, R) with *lg *of 1. This is further confirmed by the three dimensional structure shown in Figure 2D. As indicated by the figure, there is no descriptor for the four top most discriminative amino acid pairs that occur in the non DNA-binding regions, and all the five occurrences are within the one DNA-binding region. Furthermore, the figure showed that the pair(R, R) with *lg *of 1and pair(P, R) with *lg *of 3 are very closed to the three dimensional structure of DNA, indicating that these two descriptors are very discriminative for DNA and protein interaction.

### Comparison with existing PSSM based encoding schemes

In this section, four protein encoding schemes based on PSSM are introduced for a comparison. They are the average score of the residues with respect to the column of certain AA type called AvePscore-20 [21], the average score of the residues of certain AA type with respect to the column of certain AA type called AvePscore-400 [74], the percentile value of the PSSM scores along with the column of certain AA type according to percent thresholds called Pscore-100 [75], and auto-correlation coefficient (ACC) transformation that can transform the PSSMs of different lengths into fixed-length vectors by measuring the correlation between two scores separated by a distance of *lg *along the sequence [76], respectively. Table 2 lists the predictive results of the proposed protein representation and other four considered protein representations on the benchmark dataset using jackknife validation.

**Table 2:** Results on benchmark dataset of different PSSM based encoding schemes through jackknife validation.

Methods	Acc(%)	MCC	SN(%)	SP(%)	AUC(%)
AvePscore-20^a^	73.95	0.480	68.57	79.09	81.40
AvePscore-400^d^	73.58	0.470	66.47	80.36	81.50
Pscore-100^c^	73.12	0.463	72.76	73.45	80.50
ACC^d^	73.77	0.475	73.14	74.36	81.90
PSSM-DT^f^	79.96	0.622	81.91	78.00	86.50

The four protein representation methods in the front of the table are four protein encoding methods for identification of DNA-binding proteins proposed in the past. The four methods and the current method PSSM-DT are based on PSSMs property of protein sequences, but the encoding method applied by them are different. The results were got by testing on benchmark dataset through jackknife validation.

^a^results obtained by in-house implementation of AvePscore-20 [21]

^b^results obtained by in-house implementation of AvePscore-400 [21]

^c^results obtained by in-house implementation of Pscore-100 [75]

^d^results obtained by in-house implementation of ACC [76]

^f^results obtained by using PSSM-DT as protein representation

**Figure 3:**
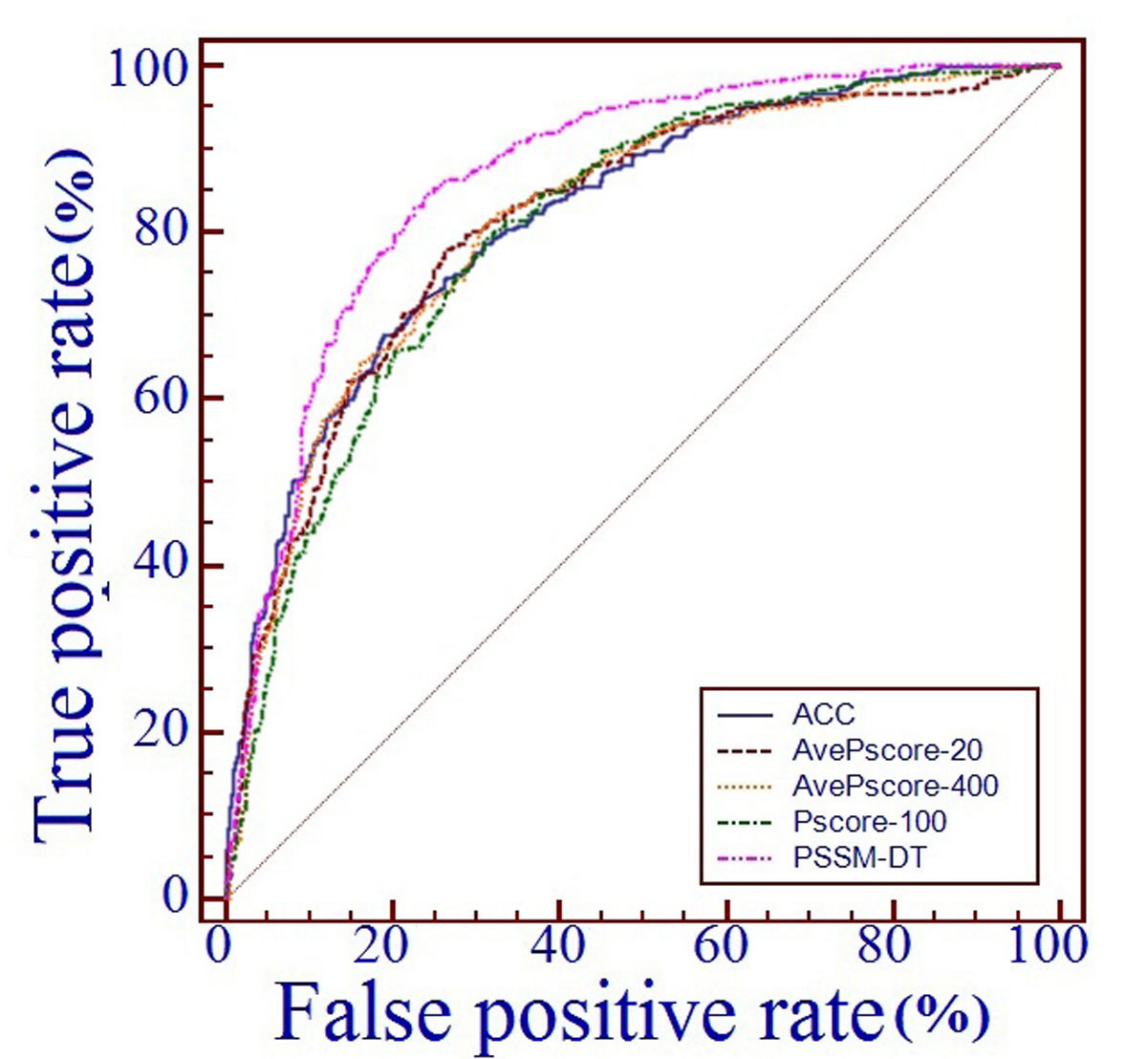
**The ROC curves of several PSSM based protein encoding methods on benchmark dataset**. The receiver operating characteristic (ROC) curves of PSSM-DT and several other existing protein encoding methods were got by testing the models on benchmark dataset through jackknife validation, where the horizontal coordinate X is for the false positive rate or 1-SP and the vertical coordinate Y is for the true positive rate or SN and a good method would yield a curve close to the coordinate (0,1) meaning low false positive rate and high true positive rate.

Furthermore, to provide a graphic illustration to show the performance of the five protein representations, the corresponding ROC (receiver operating characteristic) curves were drawn in Figure 3, where the horizontal coordinate X is for the false positive rate or 1-SP and the vertical coordinate Y is for the true positive rate or SN. The best method would yield a point with the coordinate (0,1) meaning 0 false positive rate and 100% true positive rate. Therefore a perfect classification method would give a point with the coordinate (0,1) and a completely random guess would give a point along the diagonal from point (0,0) to (1,1). The area under the ROC curve called AUC is often used to indicate the performance quality of binary classification methods, where the larger the area, the better the predictive quality is.

As shown in Table 2 and Figure 3, the PSSM-DT based protein representation generated the highest performance and outperformed the other four protein representations based on PSSM, indicating that PSSM-DT based protein representation is effective for DNA-binding protein identification.

### Comparison with existing prediction methods

**Figure 4:**
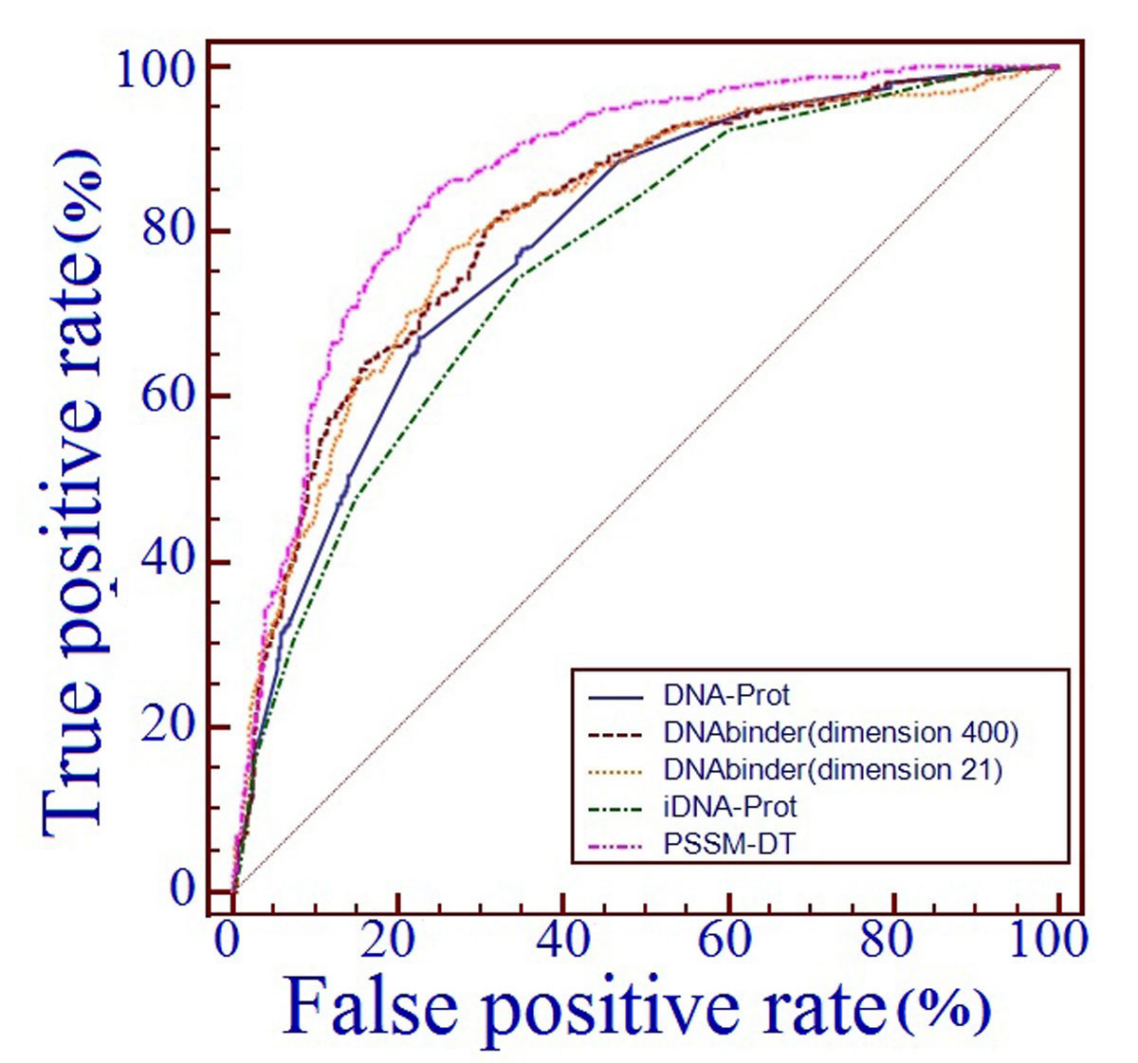
**The ROC curves of several predictive methods on benchmark dataset**. The receiver operating characteristic (ROC) curves of SVM-PSSM-DT and several other existing DNA-binding protein predictors were got by testing the models on benchmark dataset through jackknife validation, where the horizontal coordinate X is for the false positive rate or 1-SP and the vertical coordinate Y is for the true positive rate or SN and a good method would yield a curve close to the coordinate (0,1) meaning low false positive rate and high true positive rate.

Table 3 shows the predictive results of SVM-PSSM-DT and four other state-of-the-art methods on the benchmark dataset through jackknife validation, including DNAbinder(dimension 21) [21], DNAbinder(dimension 400) [21], DNA-Port [74] and iDNA-Prot [16]. DNAbinder(dimension 21) and DNAbinder(dimension 400) encode features from their PSSM based evolutionary information and utilize SVM to build prediction model. iDNA-Prot applies grey model to integrate the features from protein sequence into the general form of pseudo amino acid composition and then inputs into a Random Forest classifier. DNA-Prot is a Random Forest classifier based on the amino acid composition, predicted second structure and some physicochemical properties. The ROC curves of the proposed method and the four predictive methods are shown in Figure 4.

**Table 3:** Results on benchmark dataset of different predictors through jackknife validation.

metric	ACC (%)	MCC	SN (%)	SP (%)	AUC(%)
DNAbinder(dimension 21)^a^	73.95	0.480	68.57	79.09	81.40
DNAbinder(dimension 400)^b^	73.58	0.470	66.47	80.36	81.50
DNA-Prot^c^	72.55	0.440	82.67	59.76	78.90
iDNA-Prot^d^	75.40	0.500	83.81	64.73	76.10
PSSM-DT^f^	79.96	0.622	81.91	78.00	86.50

The four methods in the front of the table are four state-of-the-art predicting methods for identification of DNA-binding proteins proposed in the past and were demonstrated to have good performance. The results of the four existing methods and SVM-PSSM-DT were got by testing on benchmark dataset through jackknife validation.

^a^results obtained by in-house implementation of DNAbinder [21]

^b^results obtained by in-house implementation of DNAbinder [21]

^c^results obtained by in-house implementation of DNA-Prot [74]

^d^results obtained by in-house implementation of iDNA-Prot [16]

^f^results obtained by using PSSM-DT as protein representation

From Table 3 and Figure 4 we can see that SVM-PSSM-DT achieved the best performance with ACC of 79.96%, MCC of 0.62 and AUC of 86.50%, outperforming other four methods by 4.56-7.41% in terms of ACC, 0.12-0.18 in terms of MCC and 5-10.4% in terms of AUC. It indicates that PSSM-DT can advance the prhedictive performance of DNA-binding proteins identification from PSSM based sequence information.

### Independent test

In order to further compare the predictive performance of SVM-PSSM-DT with other existing methods, we evaluated the proposed method on the independent dataset PDB186. It was recently constructed by Lou et al [75] to validate the quality of predictions, which consists 93 DNA-binding proteins and equal number of non DNA-binding proteins selected from PDB. Since there are some sequences from the benchmark dataset that shared high sequence identity with the independent dataset PDB186, the tool CD-HIT [77] was applied to remove the sequences from the benchmark dataset having more than 25% sequence identity to any one in a same subset in the independent dataset PDB186 to avoid homology bias. Table 4 lists the predictive results of the proposed method and several relevant existing methods, including iDNA-Prot [16], DNA-Prot [74], DNAbinder [21], DNABIND [34], and DNA-Threader [78], to our best knowledge.

**Table 4:** Results on Independent dataset PDB186 of different predictors^a^

Methods	Acc(%)	MCC	Sn(%)	Sp(%)	AUC(%)
iDNA-Prot	67.20	0.344	67.70	66.70	83.30
DNA-Prot	61.80	0.240	69.90	53.80	79.60
DNAbinder	60.80	0.216	57.00	64.50	60.70
DNABIND	67.70	0.355	66.70	68.80	69.40
DNA-Threader	59.70	0.279	23.70	95.70	N/A
DBPPred	76.90	0.538	79.60	74.20	79.10
PSSM-DT	80.00	0.647	87.09	72.83	87.40

The six methods in the front of the table are six useful predicting methods for identification of DNA-binding proteins proposed in the past and were demonstrated to have good performance. The results of the six existing predicting methods and the SVM-PSSM-DT were achieved on the dataset PDB186 by their model trained on benchmark dataset.

^a^The results of iDNA-Prot [16], DNA-Prot [74], DNAbinder[21], DNABIND [34], DNA-Threader [78] and DDPPred [75] were obtained from [75].

**Figure 5:**
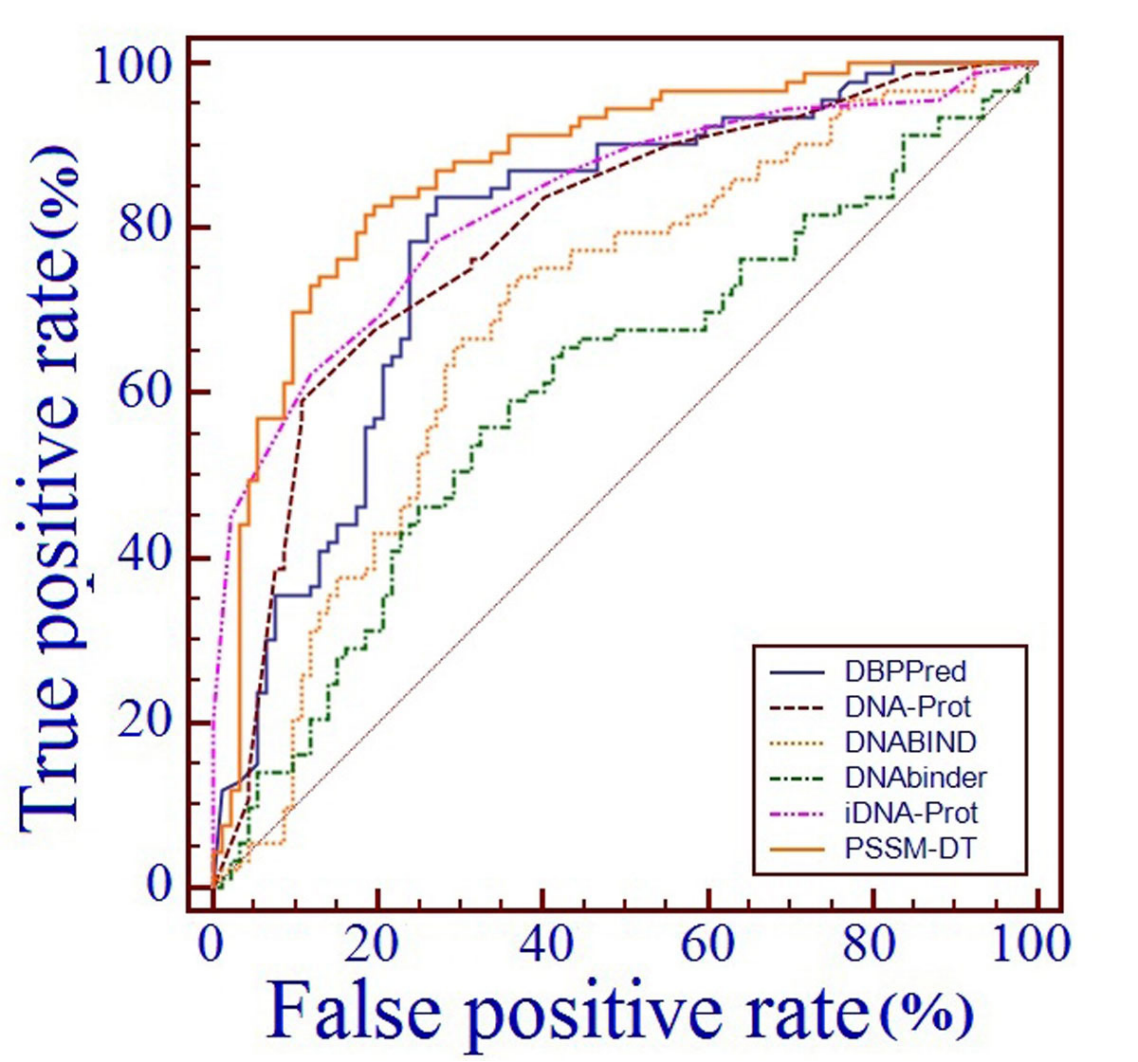
**The ROC curves of several predictive methods on Independent dataset**. The receiver operating characteristic (ROC) curves of SVM-PSSM-DT and several other existing DNA-binding protein predictors were got by testing the models trained by benchmark dataset on independent dataset PDB186, where the horizontal coordinate X is for the false positive rate or 1-SP and the vertical coordinate Y is for the true positive rate or SN and a good method would yield a curve close to the coordinate (0,1) meaning low false positive rate and high true positive rate.

Moreover, to provide a graphic illustration to show the performance comparisons of the SVM-PSSM-DT with other existing state-of-the-art predictors, the corresponding ROC curves were drawn in Figure 5. The experimental real value results of three predictors are provided by [75], including DBPPred [75], DNAbinder [21] and DNABIND [23]. And the real value outputs of the proposed method, iDNA-Prot and DNA-Prot are obtained by testing their predictors trained on benchmark dataset on independent dataset PDB186.

From Table 4 and Figure 5 we can see that among the seven predictive methods, the proposed method has the highest performance with ACC of 80.00%, MCC of 0.674 and AUC of 87.40% and DBPPred is the known reported predictive method with the best predictive performance (ACC = 76.90%, MCC = 0.538 and AUC = 79.10%). So the independent prediction of SVM-PSSM-DT is improved by ACC of 3.105%, MCC of 0.136 and AUC of 8.30% when compared with the DBPPred method, indicating that SVM-PSSM-DT is an effective prediction model for DNA-binding protein identification.

### Web-server guide

We have constructed a user-friendly web-server of SVM-PSSM-DT freely accessible to the public. Moreover, for the convenience of the vast majority of experimental scientists, a step-by-step guide is provided below on how to use the web-server to get the desired results.

**Figure 6:**
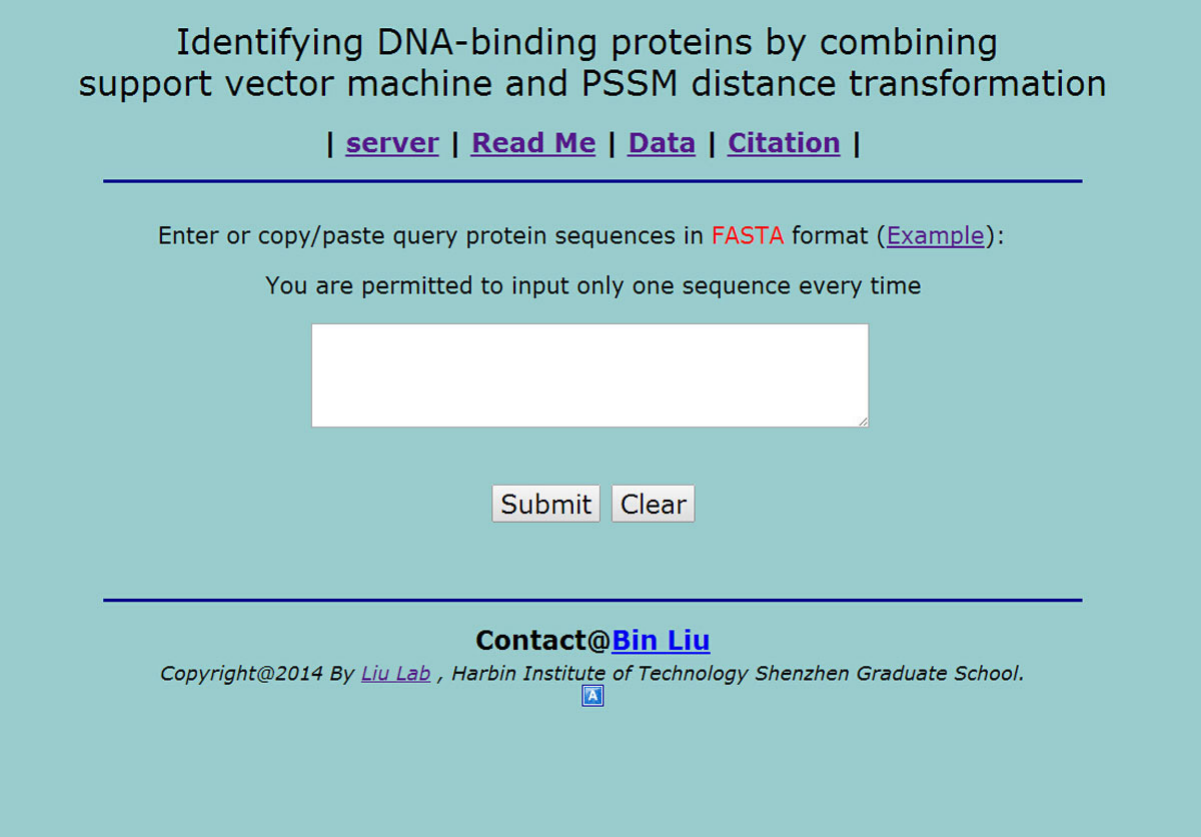
**The top page of the web-server**. In the top page, you can type or copy and paste the query protein sequences into the input box at the center, obtain the brief introduction about this web-server by clicking on Read Me button and see information about FASTA format by clicking on the Example button right above the input box.

**Step 1**. Open the web-server by clicking the link [79] and you will see the home page as shown in Figure 6. Click on the Read Me button you can obtain the brief introduction about this web-server.

**Step 2**. Either type or copy and paste the query protein sequences into the input box at the center of Figure 6. AS this server need calculate the PSSM profile for every protein sequence through PSI-BLAST, which is a time-consuming operation, thus it receive only a query protein sequence at a time. The input sequence should be in the FASTA format and example sequences in FASTA format can be seen by clicking on the Example button right above the input box.

**Step 3**. Click on the Submit button to submit the query sequence to the server, then you will see the predicted results on your screen. For example, use the protein 1IGN chain B as a query sequence, you will see on your screen that the predictive result is "DNA-binding protein".

## Conclusion

In this work, we investigated the idea of identifying DNA-binding proteins from sequence by combining SVM and PSSM-DT. The PSSM-DT is the features from PSSM by considering the probabilities of pairs of amino acid separated by certain number of sites along the sequence in a sequence. A benchmark test on a dataset of 525 DNA-binding proteins and 550 proteins which do not bind to DNA using jackknife validation showed that SVM-PSSM-DT achieved the best predicting performance with ACC of 79.96%, MCC of 0.62 and AUC of 86.50%, and performed better than other state-of-the-art methods by 4.56-7.41% in terms of ACC, 5-10.4% in terms of AUC and 0.12-0.18 in terms of MCC. Subsequently, the blind test performed on the Independent dataset PDB186 indicated that the proposed predictive method obtain an ACC of 80.00%, MCC of 0.647 and AUC of 87.40%, and outperformed some existing state-of-the-art methods. Additionally, the discriminant weight of the descriptors in PSSM-DT-based protein representation is calculated based on the benchmark dataset and the analysis results show that pair(R, R), pair(R, P), pair(P, R) and pair(A, R) are the top four most discriminative amino acid pairs. The three dimensional structure of the protein 1AKH chain A showed that the descriptors for the top four most discriminative amino acid pairs only occur in the DNA-binding regions of the protein, indicating that PSSM-DT is a useful tool for identifying DNA-binding protein.

## Availability of supporting data

The data set supporting the results of this article is included within the article and its additional file 1.

## Competing interests

The authors declare that they have no competing interests.

## Authors' contributions

BL conceived of the study and carried out the DNA binding protein study, participated in designing the study, coding the experiments, drafting the manuscript and performing the statistical analysis. JYZ participated in coding the experiments and drafting the manuscript. RFX, YLH, HPW, XLW participated in performing the statistical analysis. All authors read and approved the final manuscript.

## Supplementary Material

Additional file 1 **benchmark dataset S**. It contains 1075 protein sequences, which are classified into subset with 525 DNA-binding proteins (positive samples) and subset with 550 non-DNA-binding proteins (negative samples). Both the accession identifier of PDB (Protein Data Bank) and sequences are given. Click here for file
